# Chemically Crosslinked Bispecific Antibodies for Cancer Therapy: Breaking from the Structural Restrictions of the Genetic Fusion Approach

**DOI:** 10.3390/ijms21030711

**Published:** 2020-01-21

**Authors:** Asami Ueda, Mitsuo Umetsu, Takeshi Nakanishi, Kentaro Hashikami, Hikaru Nakazawa, Shuhei Hattori, Ryutaro Asano, Izumi Kumagai

**Affiliations:** Department of Biomolecular Engineering, Graduate School of Engineering, Tohoku University, 6-6-11 Aoba, Aramaki, Aoba-ku, Sendai 980-8579, Japan; asamiueda.bj@gmail.com (A.U.); nakanishi@bioa.eng.osaka-cu.ac.jp (T.N.); kentarou.hashikami@takeda.com (K.H.); hikaru@tohoku.ac.jp (H.N.); shuhei.hattori.t1@dc.tohoku.ac.jp (S.H.); ryutaroa@cc.tuat.ac.jp (R.A.)

**Keywords:** antibody, bioinformatics, chemical conjugation

## Abstract

Antibodies are composed of structurally and functionally independent domains that can be used as building blocks to construct different types of chimeric protein-format molecules. However, the generally used genetic fusion and chemical approaches restrict the types of structures that can be formed and do not give an ideal degree of homogeneity. In this study, we combined mutation techniques with chemical conjugation to construct a variety of homogeneous bivalent and bispecific antibodies. First, building modules without lysine residues—which can be chemical conjugation sites—were generated by means of genetic mutation. Specific mutated residues in the lysine-free modules were then re-mutated to lysine residues. Chemical conjugation at the recovered lysine sites enabled the construction of homogeneous bivalent and bispecific antibodies from block modules that could not have been so arranged by genetic fusion approaches. Molecular evolution and bioinformatics techniques assisted in finding viable alternatives to the lysine residues that did not deactivate the block modules. Multiple candidates for re-mutation positions offer a wide variety of possible steric arrangements of block modules, and appropriate linkages between block modules can generate highly bioactive bispecific antibodies. Here, we propose the effectiveness of the lysine-free block module design for site-specific chemical conjugation to form a variety of types of homogeneous chimeric protein-format molecule with a finely tuned structure and function.

## 1. Introduction

Antibodies are composed of several structurally and functionally independent domains. Therefore, they can be fragmented and their block modules can be rearranged to form recombinant proteins with non-native structures and functions [1]. The variable region fragment (Fv), which are composed of variable regions of heavy chain (VH) and light chain (VL), has the antibody’s antigen-binding function, and the single chain Fv (scFv) where VH domain is linked with VL domain via a short peptide is sufficiently small for it to be used in recombinant genetic fusion approaches for pharmaceutical [2,3], diagnostic [4], and biosensor [5] studies. In the pharmaceutical field, antigen-binding modules have been fused with functional proteins—including peptides [6], cytokines [7,8], enzymes [9], and toxic proteins [10]—to form bifunctional targeted therapeutic molecules.

Bispecific antibodies with two different Fvs have been constructed from two distinct monoclonal antibodies, and they have been used to induce interactions between two types of cell by forming linkages between the two target antigens on the surfaces of the cells [11,12,13,14]. In particular, the crosslinking of highly cytotoxic immune cells, such as T-cells, to cancer cells induces the immune cells to damage the cancer cells [11,12,13,14]. First-generation bispecific antibodies were produced by means of hybrid hybridomas or by chemical crosslinking from wild-type monoclonal antibodies, but both of these approaches generated populations of antibody molecules with heterogeneous structural properties [15,16,17]. Advances in recombinant genetic fusion approaches have enabled the production of homogenous bispecific antibody molecules, and various bispecific antibodies have been genetically designed with antibody fragments as block modules [18]. However, the genetic fusion approaches restrict the conjugation sites to the termini of each block module, so the variety of possible quaternary structures is limited. In the case of T-cell-activating bispecific antibodies, several structural formats for the Fvs have been reported (for example, diabody, single-chain diabody, tandem single-chain, and BiBian), and changing the structural formats can cause an approximately 1000-fold difference in cytotoxicity [19,20,21], indicating the importance of quaternary structures on the functions of bispecific antibodies. Crosslinking techniques, which provide a wider variety of steric arrangements of block modules, would enable us to find appropriate linkages between the block modules of Fvs to generate functional bispecific antibodies.

In this study, we propose a method of site-specific chemical conjugation for constructing a variety of homogeneous bivalent and bispecific antibodies by the combination of mutation and chemical conjugation techniques. Although several functional groups of amino acid side chains have been used for chemical conjugation with coupling reagents [22], the thiol group in cysteine is the most generally applied for site-specific conjugation because the thiol group exists only in cysteine, whose frequency of appearance in proteins is lower than that of other amino acids [23,24]. However, cysteine can induce the deactivation of the proteins, especially for proteins in which cysteines are important for their structure and function. The immunoglobulin-fold domain in Fvs has two highly conserved cysteine residues which form an internal disulfide linkage, which is of particular importance for most proteins in the immunoglobulin superfamily because this linkage is critical to the stability of these proteins [25,26]. Therefore, mutations of cysteine in Fvs can cause incorrect disulfide linkages leading to the formation of inactive Fvs [27].

Here, we focused on lysine for site-specific chemical conjugation. Lysine, which is hydrophilic, frequently appears on the surface of proteins, and this amino group has been used as a chemical conjugation site. However, the presence of multiple lysines creates a challenge for conjugation to specific positions. In the present study, we constructed a bivalent antibody from humanized 528 (h528) Fv with affinity for the epidermal growth factor receptor (EGFR) frequently overexpressed on cancer cells [28], and a bispecific antibody from the h528 Fv and the agonistic humanized OKT3 (hOKT3) Fv that recruits CD3 receptors on T cells [29]. The targets of EGFR and CD3 has been studied for bispecific antibody which crosslinks cytotoxic immune cells to cancer cells induces the immune cells to damage the cancer cells. Here, we generated starting structures without lysine residues in order to enable the arrangement of block modules in various directions and then returned the residue to lysine at the site where the block module was to be conjugated. Molecular evolution, crystal structure, and bioinformatics techniques assisted us in finding alternative amino acids for the lysine residues so that the lysine-free Fv variants were generated without inactivation. The re-mutation to lysine in the lysine-free variants enabled the formation of homogeneous bivalent and bispecific antibodies with novel arrangements of block modules that cannot be formed by only gene fusion techniques. Here, we present the potential of combining mutation and chemical conjugation techniques in the design of homogeneous chimeric antibody with a finely tuned structure and function.

## 2. Results

### 2.1. Mutation of Lysine Residues in h528 Fv

**Figure 1:**
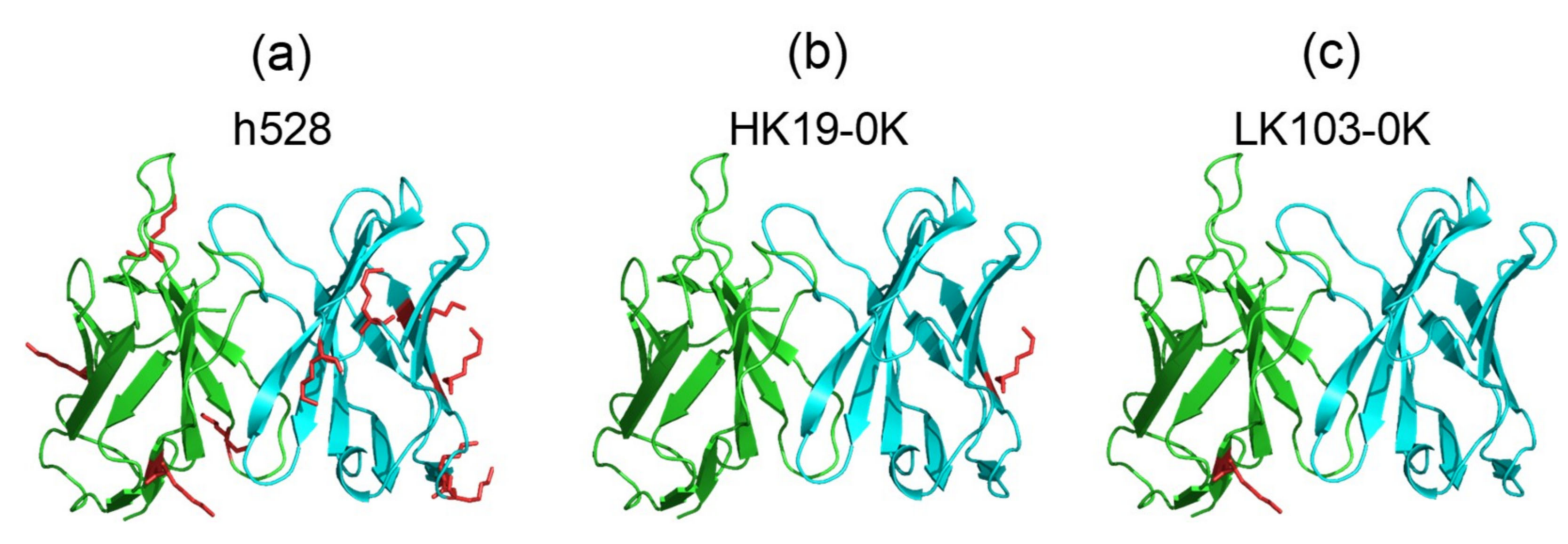
Lysine positions on (**a**) h528 scFv, (**b**) HK19-0K h528 scFv where all lysines were mutated except for HK19, and (**c**) LK103 0K h528 scFv where all lysines were mutated except for LK103. Green-colored and blue-colored domains are VL and VH domains, respectively, and red-colored residues are lysine. PDB code of the used crystal structure used is 1WT5.

The h528 scFv has eleven lysine residues: three on complementarity determining region (CDR) and eight on framework region (FR). With respect to the CDR, we previously identified alternative mutations of lysine by means of phage display methods (Table 1 and Table 2) [30,31]. In the FR, the mutation data previously reported by Onda et al. [32] were applied for identifying alternative mutations of lysine (Supplementary Table S1). In the comparison with the Kabat database [33], where appearance frequency of amino acids in a huge number of reported antibody sequences can be calculated at each residue position, the amino acids which are listed in the Kabat database were selected at H12, L39, and L74, but the selected amino acids at H13, H19, L103, and L107 were not listed. Consequently, we designed a sequence for the h528 scFv without lysine in the CDR and FR (termed 0K). For the site-specific chemical conjugation, a single mutated residue in the FR of the 0K scFv was returned to lysine: the 19th residue in the VH domain and the 103rd residue in the VL domain was independently returned to lysine (termed HK19-0K scFv and LK103-0K scFv, respectively, Figure 1b,c). The HK19 and LK103 residues are in the vertical and opposite direction to create a conjugation side on the side and tail of the scFv, respectively.

The lysine-mutated scFvs were not expressed as soluble forms in *E. coli*, but they could be refolded: final yields of the refolded scFvs were 1~1.8 mg/L-media and the circular dichroism spectra were similar to that of wild-type h528 scFv (Supplementary Figure S1). In the surface plasmon resonance (SPR) measurements, 0K_CDR_ scFv had comparable affinity for the target of EGFR to that of wild-type scFv; whereas, the mutations in FR caused 5–8-fold increase of the dissociation constant (K_D_) (Table 2). The change of the affinity is attributed mainly to the decrease of association rate, probably due to charged mutation in non-paratope regions.

### 2.2. Mutation of Lysine Residues in hOKT3 Fv

The hOKT3 Fv has nine lysine residues: three in the CDR and six in the FR. With respect to the CDR, the Kabat database was applied for the mutations, because the co-crystal structure of OKT3 Fv plus CD3 showed that no lysine residues in the CDR bind to CD3 [34]: the amino acids with the highest appearance frequency or higher next to lysine in the Kabat database were selected (Supplementary Table S1). In the FR, the mutation data by Onda et al. were also applied in the same manner to h528 Fv. In the comparison with the Kabat database, the amino acids proposed from Onda’s data were those with the highest appearance frequency or higher next to lysine in the Kabat database, except for L41 and L102 (Supplementary Table S1): the proposed threonine at L41 is the second highest appearance frequency in the Kabat database and the Kabat database showed no alternative mutation at L102. Therefore, in the hOKT3 0K scFv variant without lysine, all the lysine residues except for L103 were mutated to the amino acids with the highest alternative appearance frequency or higher next to lysine in the Kabat database (Table 3).

**Figure 2:**
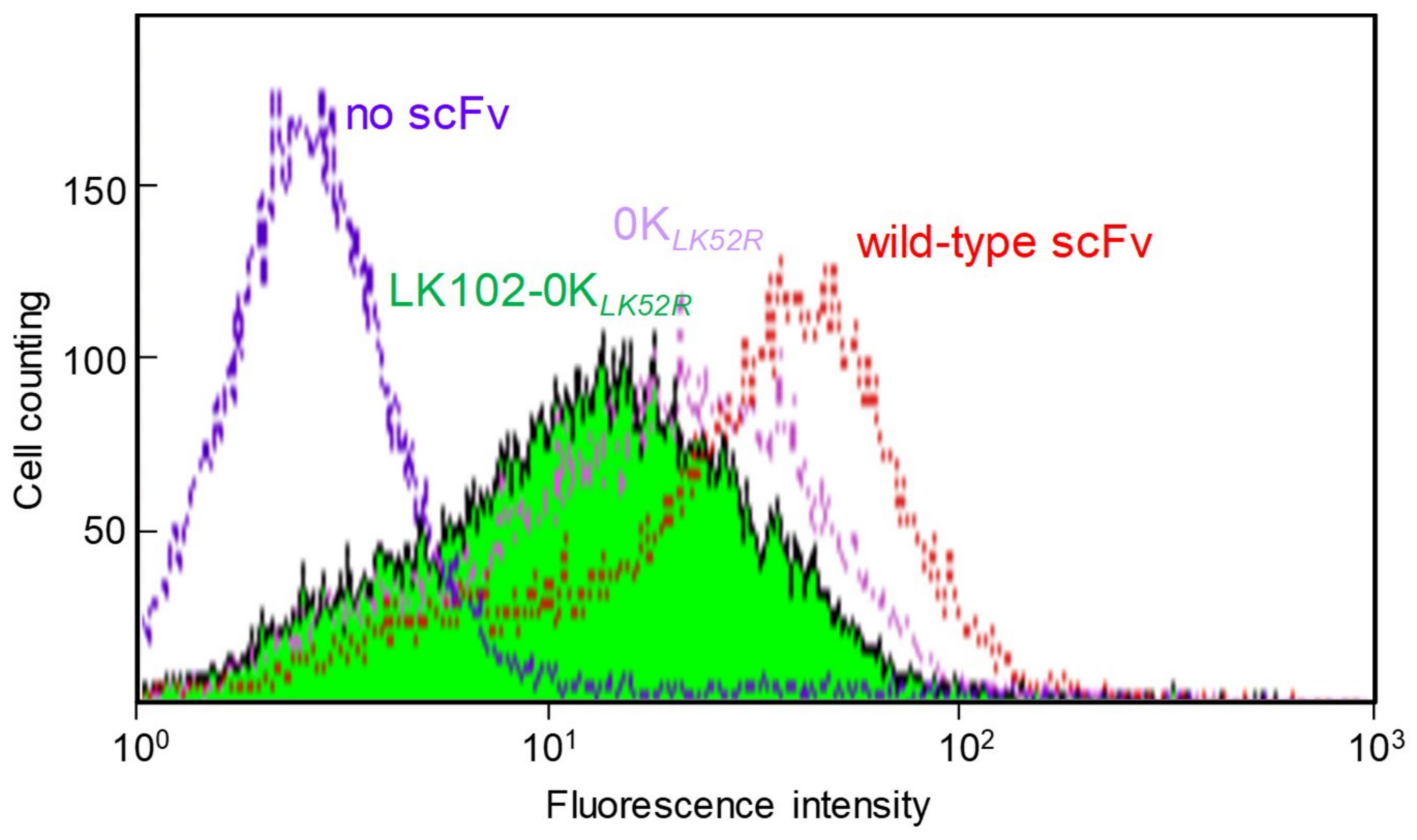
Flow cytometry histogram of T-LAK cells measured with flow cytometry. 10^6^ Cells were incubated with 0K*_LK52R_* and LK102-0K*_LK52R_* hOKT3 scFvs labeled with FITC-labeled anti-c-myc’ IgG antibody, and they were analyzed by means of flow cytometry (purple and filled green, respectively). Cells incubated with wild-type scFv and without wild-type scFv were also analyzed by FITC-labeled anti-c-myc’ IgG antibody (red and navy blue, respectively).

In the co-crystal structure of OKT3 Fv plus CD3, no lysine residues in the CDR bind to CD3, but the 52nd lysine residue in the FR of VL interacts with the 49th aspartic acid residue in the CDR of VL which forms an ion pair with the 189th lysine residue in CD3: these three amino acids can make an ion pair network. Actually, the hOKT3 0K scFv variant hardly bound to CD3. In contrast, the LK52-0K scFv variant (Table 3), in which the 52nd residue in the VL domain of the 0K scFv was returned to lysine, was the lysine-returned 0K scFv variant whose binding was most similar to that of the wild-type scFv (Supplementary Figure S2). To make the ion pair network, the LK52 residue was mutated to arginine (Table 3, 0K*_LK52R_* scFv) so that the 0K*_LK52R_* scFv bound to CD3, although the binding strength was weaker than that of the wild-type scFv (Figure 2).

**Figure 3:**
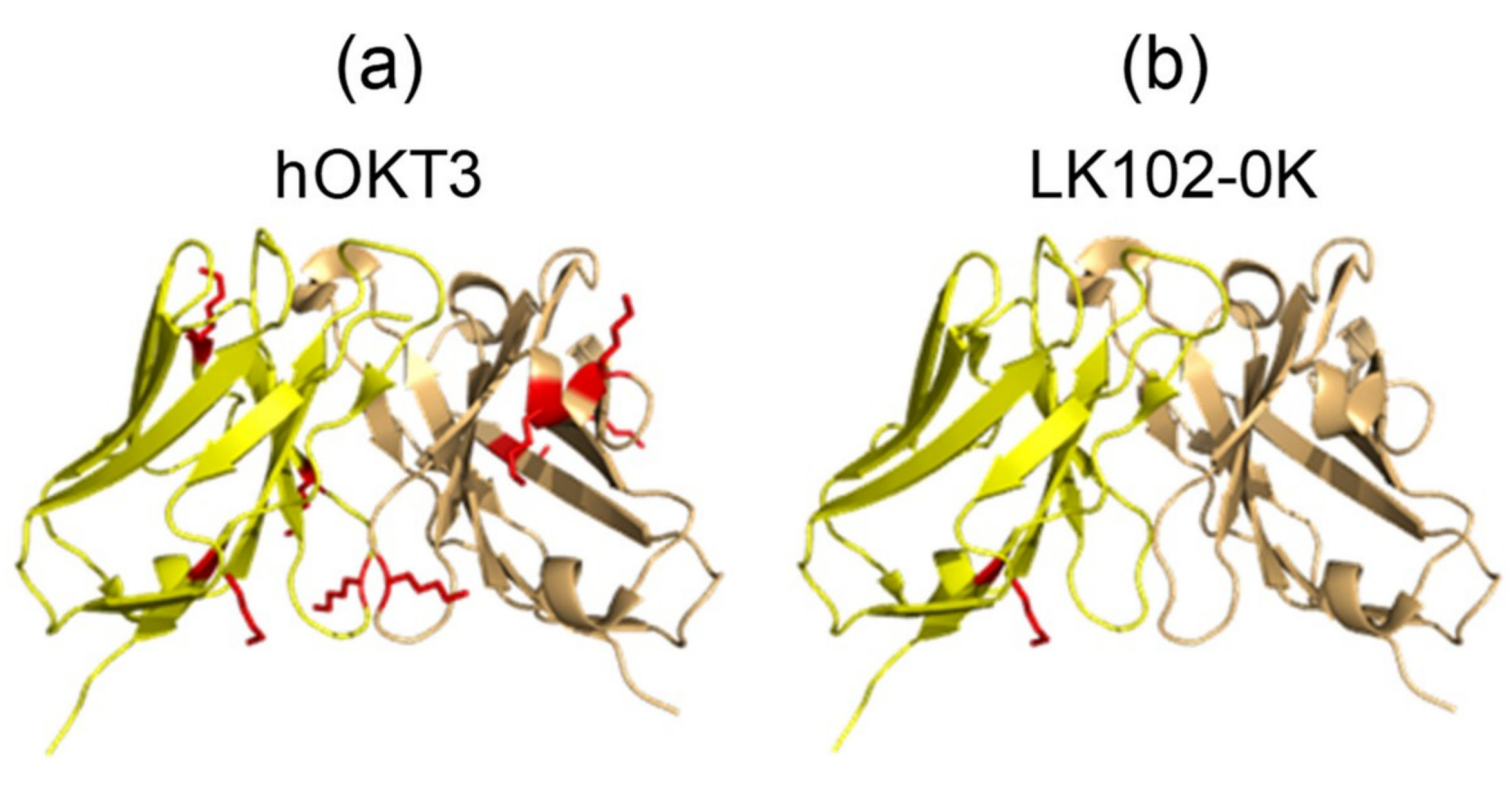
Lysine positions on (**a**) hOKT3 scFv and (**b**) LK102-0K scFv where all lysines were mutated except for LK102. Yellow-colored and wheat-colored domains are VL and VH domains, respectively, and red-colored residues are lysine. The code of the used crystal structure is 1SY6.

For the site-specific chemical conjugation, the 102nd residue in the VL domain was returned to lysine in the 0K*_LK52R_* scFv to create a conjugation site on the tail of the scFv (LK102-0K*_LK52R_* scFv, Figure 3b). The lysine-mutated scFv was expressed as an insoluble fraction, so that they were refolded: final yields of the refolded scFvs were 0.4–1.1 mg/L-media and the circular dichroism spectra were similar to that of wild-type hOKT3 scFv (Supplementary Figure S1). The refolded scFv showed binding comparable to that of 0K*_LK52R_*scFv (Figure 2).

### 2.3. Chemical Conjugation for Forming Homo scFv Dimers

**Scheme 1:**
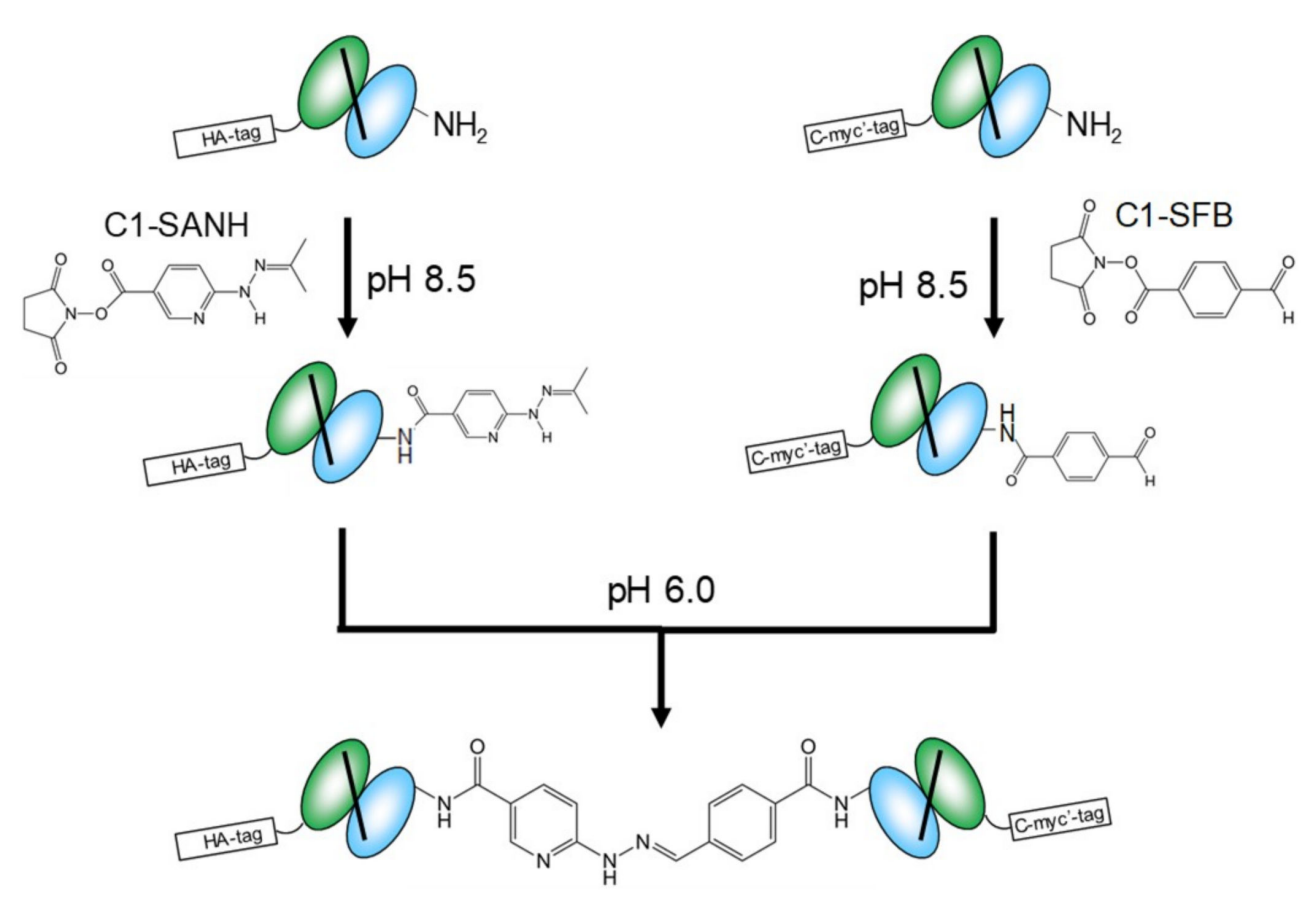
Chemical crosslinking pathway for generating a bivalent h528 scFv dimer.

**Figure 4:**
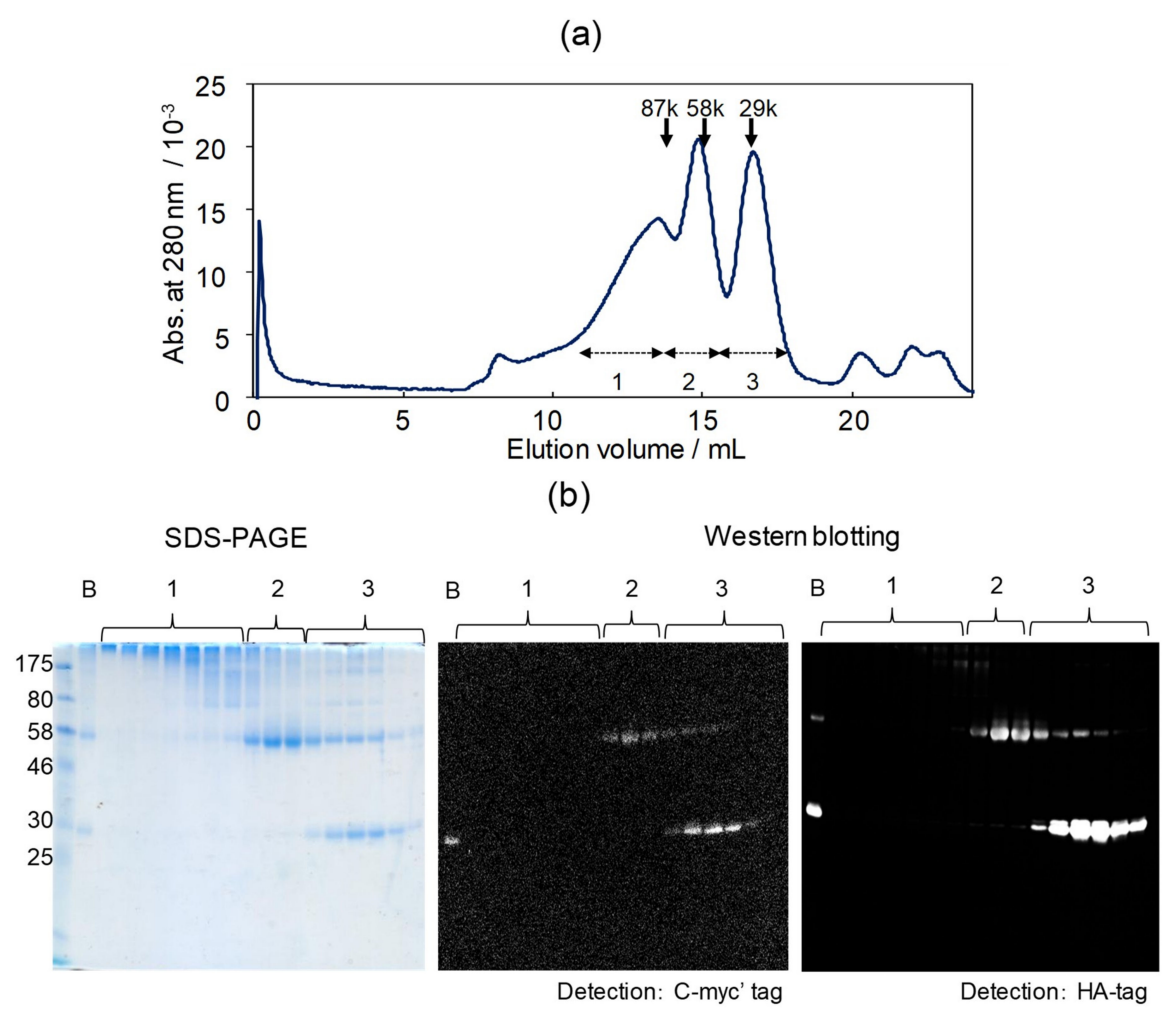
(**a**) Size-exclusion chromatography of reacted h528 HK19-0K scFvs, and (**b**) SDS-PAGE and western blotting for the reacted scFv (B) and the fractions in the chromatography (1~3). Western blotting used anti-c-myc′ antibodies conjugated with HRP (middle) and anti-HA antibodies with HRP (right).

A two-step chemical reaction is carried out to form a homo scFv dimer from lysine-mutated scFv variants (Scheme 1). In the first step, initiator molecules of hydrazine or benzoic acid (SANH or SFB; Supplementary Figure S3) are reacted with the side chains of lysine on the scFv via the coupling reaction of *N*-hydroxysuccinimide (NHS) at pH 8.5. After the removal of unreacted initiator molecules, the two chemically modified scFvs are mixed under acidic conditions so that the initiator molecules on the scFvs react to form a hydrazone bond, resulting in the formation of an scFv dimer. Here, two h528 KH19-0K scFvs with different tag-sequences, c-myc′-tag and HA-tag, were used to detect the scFv derived from each pathway in the formed dimer. The scFvs tagged with the c-myc′-tag and HA-tag were able to be concentrated to 20 μM without aggregation, and they were reacted with SANH and SFB, respectively. After the reacted scFvs were mixed, they were purified by means of size-exclusion chromatography (SEC) to fractionate the dimerized scFvs (Figure 4). In the fraction in which we expected scFv dimers to be eluted, the reacted scFvs showed dimer formation and were detected with anti-c-myc′-tag and anti-HA-tag antibodies; the chemical coupling efficiency was estimated to be ~50% from the band intensity in SDS-PAGE. Therefore, this two-step chemical reaction and SEC purification was applied to form scFv dimers with different crosslinking structures.

### 2.4. Binding of Bivalent scFv Dimers to Target-Immobilized Substrate

**Figure 5:**
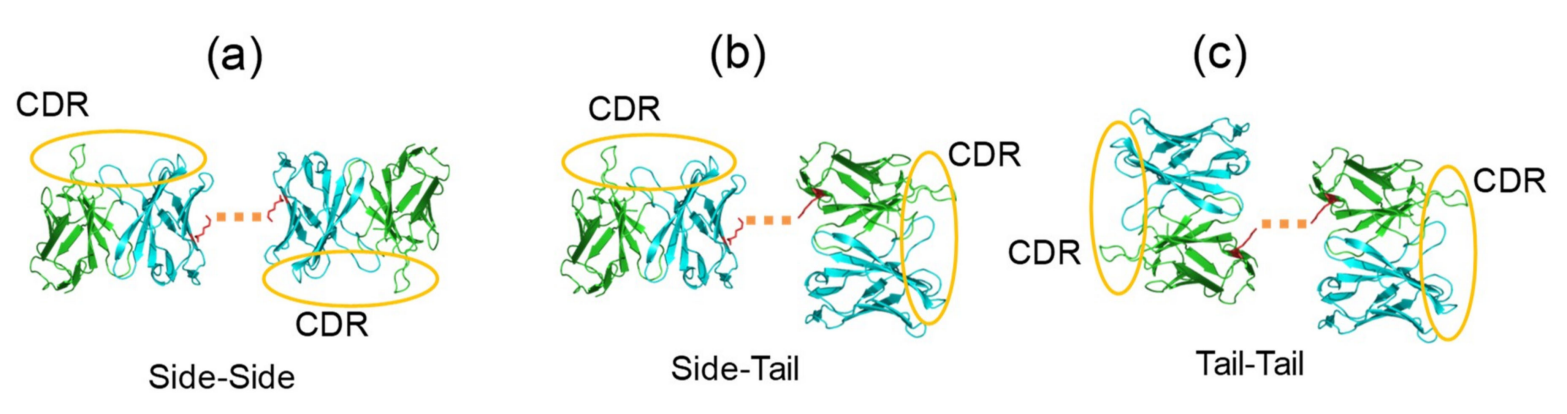
Chemically crosslinked bivalent h528 scFv dimers (VH: light blue, VL: green) with (**a**) two HK19-0K scFvs, (**b**) HK19-0K and LK103-0K scFvs, and (**c**) two LK103-0K scFvs.

**Figure 6:**
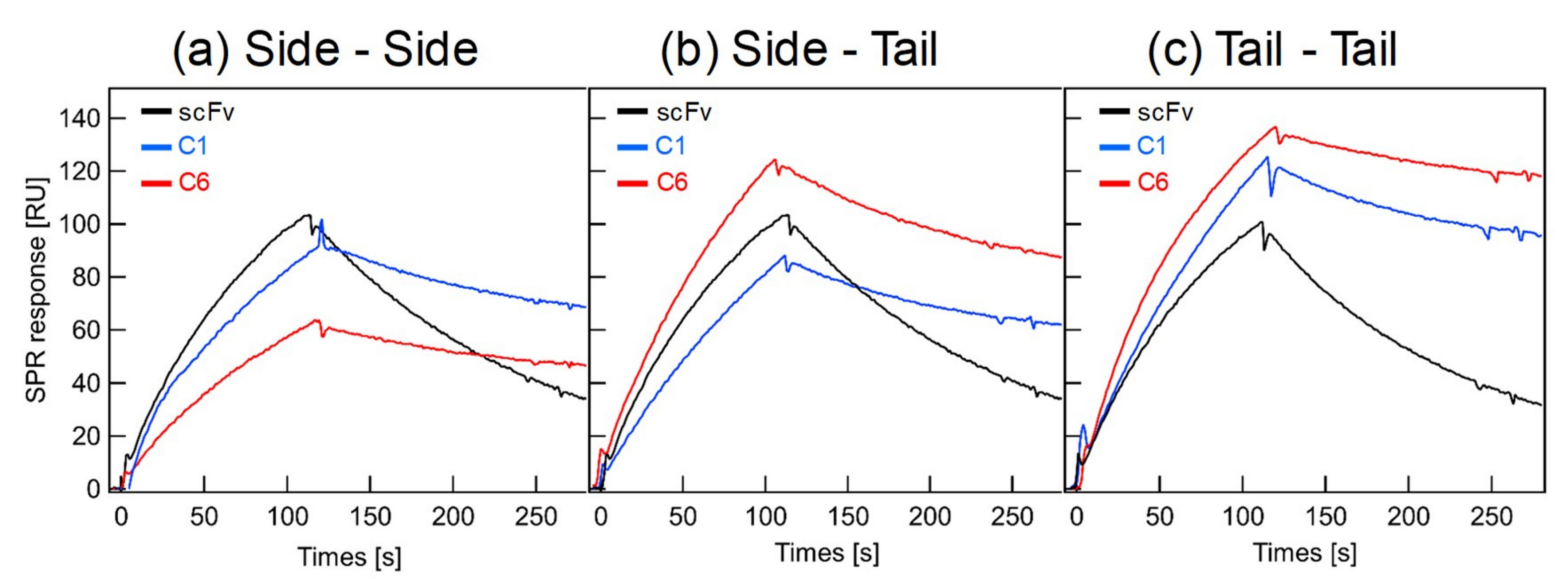
SPR sensorgrams for the binding of scFv (black curve), scFv dimer with C1 linker (blue curve), and scFv dimer with C6 linker (red curve), with respect to the immobilized EGFR (RU: 2605~2833). (**a**) side–side scFv at the concentration of 140 nM (C1) and 110 nM (C6), (**b**) side–tail dimers at the concentration of 115 nM (C1) and 130 nM (C6), (**c**) tail–tail dimers at the concentration of 140 nM (C1) and 165 nM (C6). HK19-0K was applied for monomeric scFv at the concentration of 125 nM in (**a**,**b**), and LK103-0K scFv at the concentration of 125 nM was applied in (**c**).

To analyze the influence that the direction of the linkage in the scFv dimers had on the binding affinity for targets, we created three dimeric formats (Figure 5): a side–side form from two HK19-0K scFvs, a side–tail form from HK19-0K and LK103-0K scFvs, and a tail–tail form from two LK103-0K scFvs. For the three scFv dimers, the linkage lengths were also varied by changing the length between the succinimide and the initiator side groups (hydrazine or benzoic acid) in the initiator molecules (C1, C6; Supplementary Figure S3). In the SPR sensorgrams for the binding to immobilized EGFR on sensorchip, all the dimers showed less dissociation than monomeric form (Figure 6): increasing the number of fragments binding to the substrate resulted in less dissociation from the target-immobilized substrates than was seen for monomeric scFvs, which is the avidity effect. This lower dissociation was prominent at highly immobilized EGFR (Table 4), supporting the avidity effect. 

In the comparison between three dimeric formats, tail–tail forms showed a significant avidity effect, while side–tail forms had comparable *K*_D_ values of the monomeric scFv, and the long C6 linker promoted association and suppressed dissociation in the tail–tail form. These results indicate that the direction of crosslinking had more influence on avidity effect and that longer linkers possibly provide a more beneficial steric situation.

### 2.5. Binding of Bivalent scFv Dimers to Target-Displaying Cells

**Figure 7:**
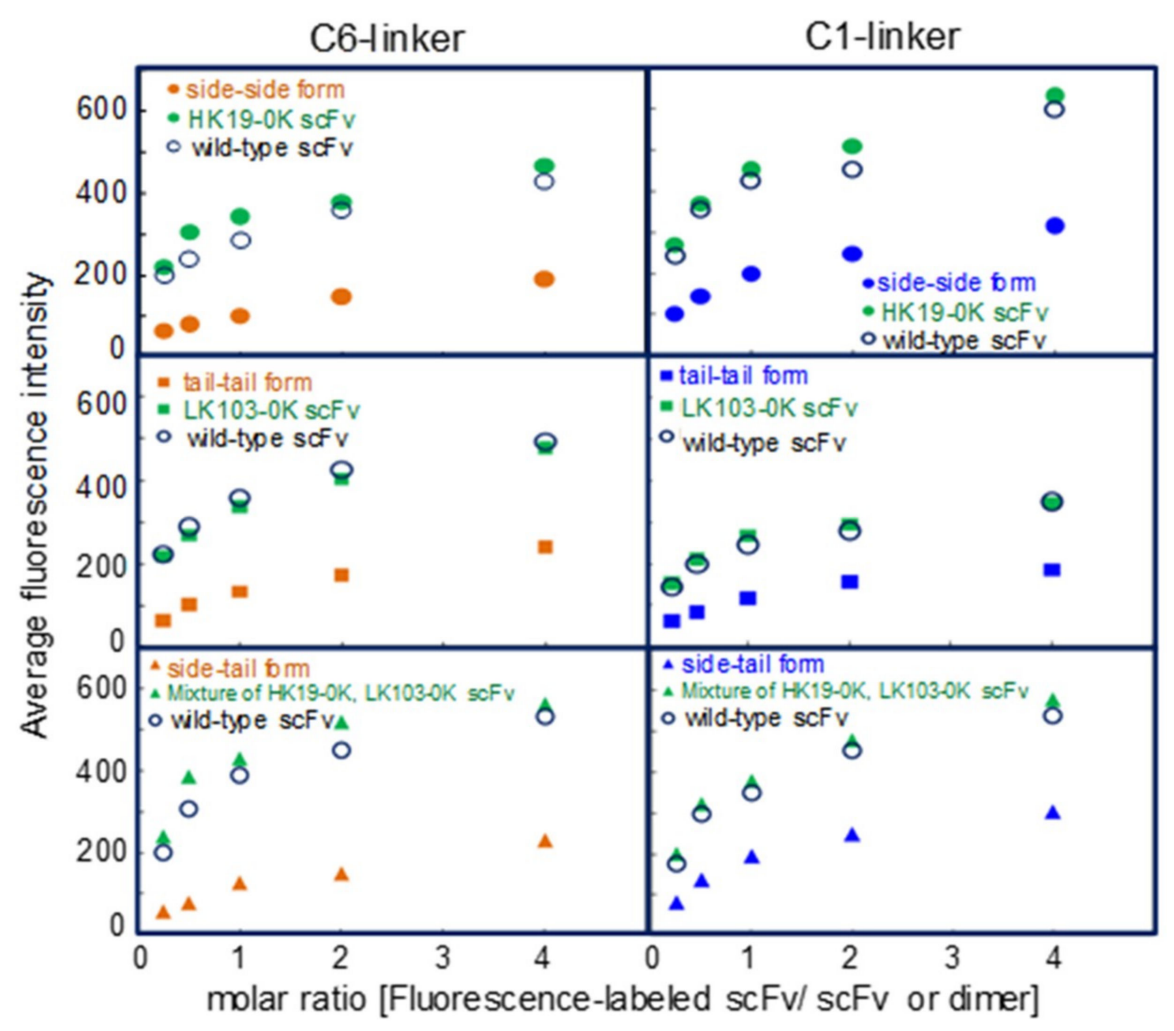
Competitive binding assay of non-labeled scFv, HK19-0K or LK103-0K scFv, side–side dimers with C1 and C6 linkers, side–tail dimers, and tail–tail dimers with C1 and C6 linkers, with FITC-labeled wild-type scFv. 10^6^ A431 cells were mixed with the non-labeled scFv or dimer (50 pmol), washed, and various amounts of fluorescently labeled wild-type scFv were added.

To analyze the avidity effect with respect to cell surfaces, a competitive binding assay was performed by means of flow cytometry (Figure 7). In this assay, non-labeled lysine-mutated scFv monomers or dimers were first allowed to bind to target-expressing cells. Then, various amounts of fluorescently labeled wild-type scFv were added and the cells bearing the labeled scFvs were counted. The competitive binding of labeled wild-type scFv in the presence of monomeric HK19-0K scFv and LK103-0K scFv was similar to that in the presence of wild-type scFv under all conditions. In contrast, in the presence of the scFv dimers, labeled wild-type scFv scarcely bound to target-expressing cells, such that the amount of bound labeled competitor was only 0.3–0.4 times that of bound wild-type scFv (Supplementary Figure S4). Therefore, the bivalent format formed by chemical crosslinking also enhanced the binding to target-expressing cells. All the scFv dimers showed comparable competitive binding, although a long linker length between scFvs in a dimer slightly promoted the binding. Considering that the difference in dissociation property is dominant in the competitive binding assay, these binding behaviors support the results of *k*_off_ for target-immobilized substrates.

### 2.6. scFv Dimers Bispecific Towards Cancer and T-Cells

**Figure 8:**
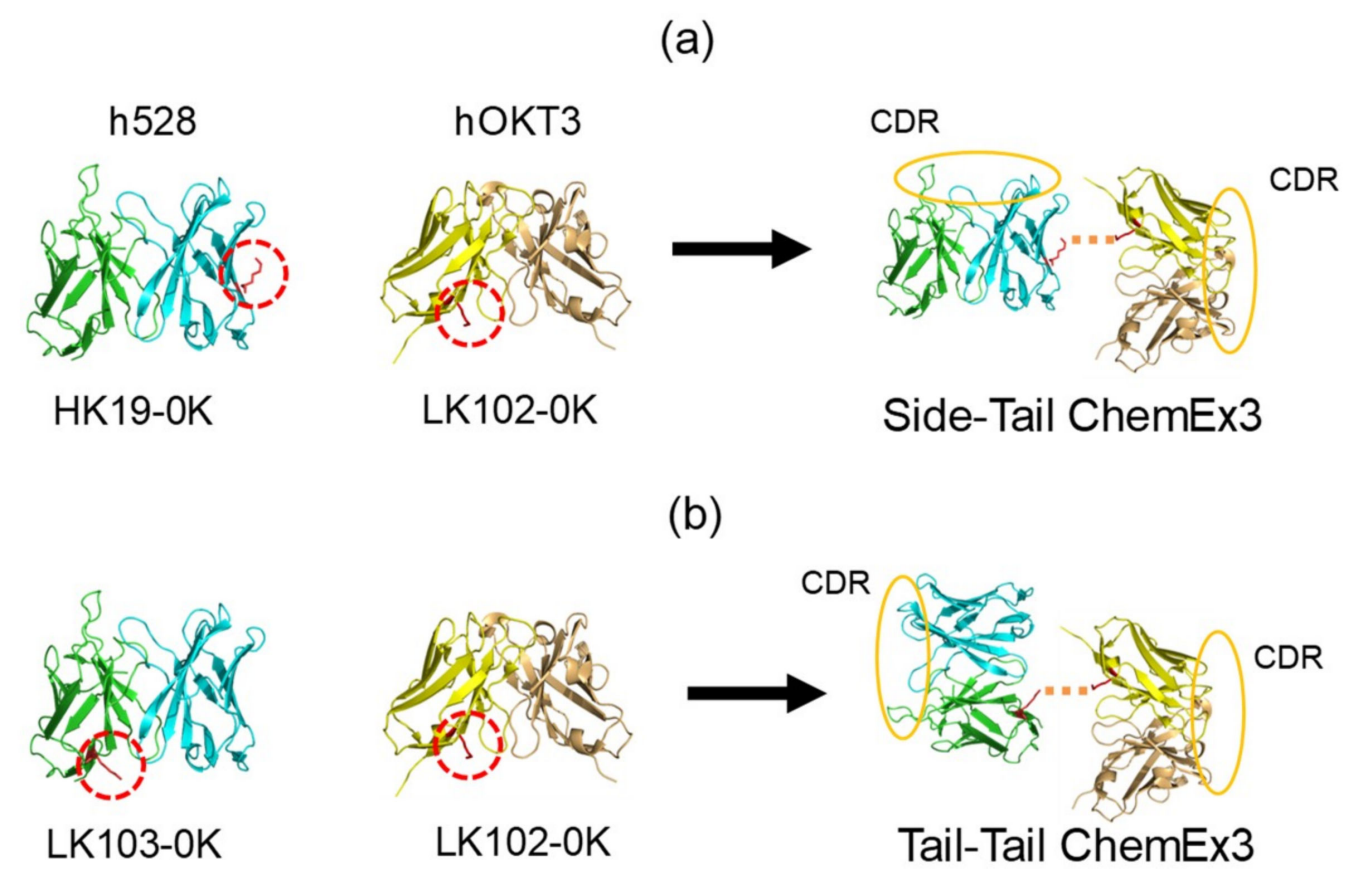
Chemically crosslinked Ex3 bispecific scFv dimers composed of (**a**) HK19-0K and LK102-0K*_LK52R_* scFvs, and (**b**) LK103-0K and LK102-0K*_LK52R_* scFvs.

To analyze the influence that the crosslinking format of the scFv dimer has on cytotoxicity, two bispecific scFv dimers with affinity for EGFR and CD3 (chemEx3 dimer) were formed: a side–tail form from h528 HK19-0K scFv and hOKT3 LK102-0K*_LK52R_* scFv, and a tail–tail form from h528 LK103-0K scFv and hOKT3 LK102-0K*_LK52R_* scFv (Figure 8). For the chemEx3 dimer, the linkage length was also varied (C1 or C6 linker). In the measurements of SPR and flow cytometry, the chemEx3 dimers with a C6 linker had comparable target binding affinities to those of unreacted h528 and hOKT3 scFvs, but the use of C1 linker caused a decrease of the binding to EGFR (Table 1 and Table 5, and Supplementary Figure S5).

**Figure 9:**
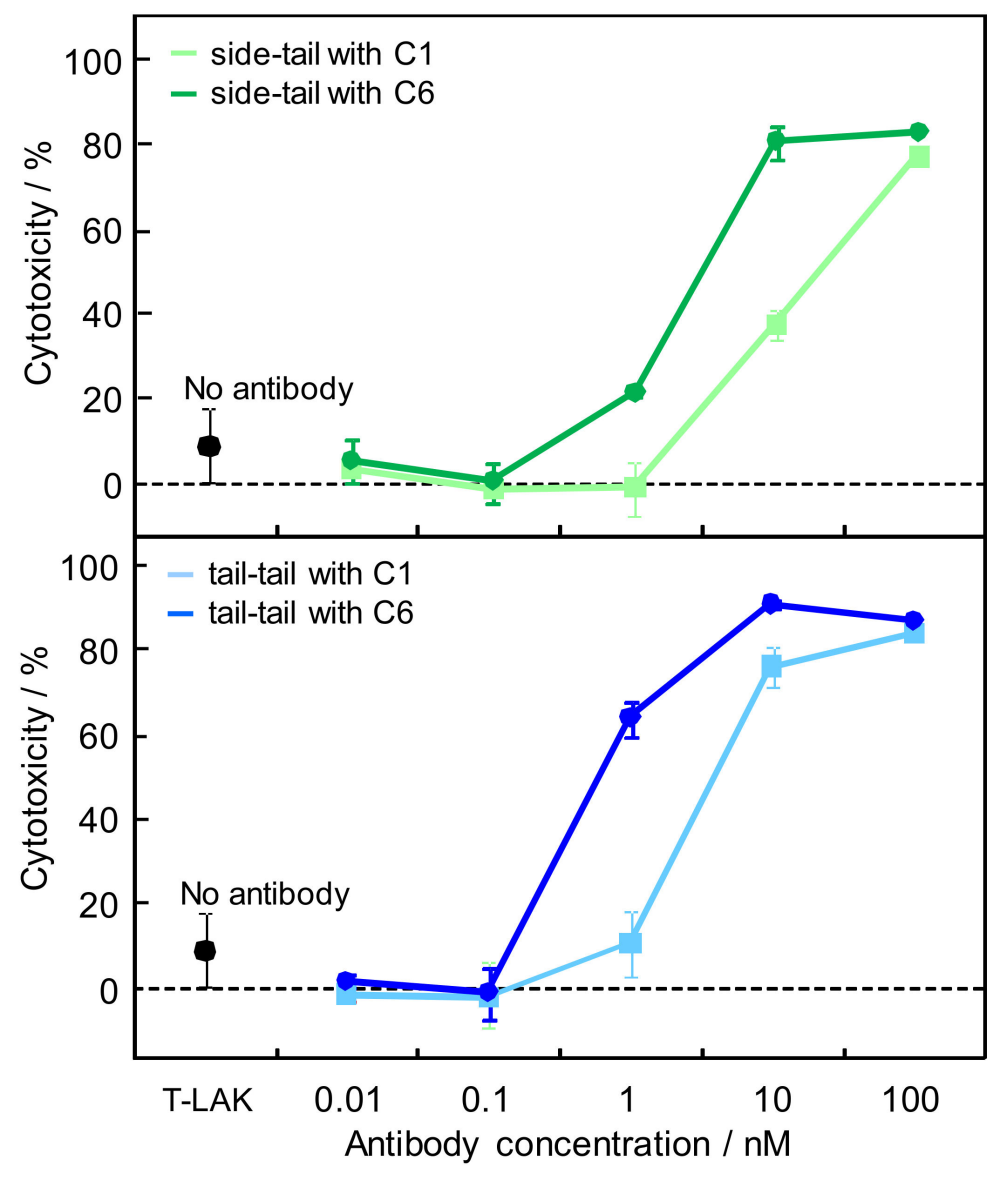
MTS assay of side–tail chemEx3 dimer with C1 and C6 linker (light green squares and green circles, respectively), tail–tail chemEx3 dimer with C1 and C6 linker (light blue squares and blue circles, respectively) for T-LAK and TFK-1 cells. On the horizontal axis, T-LAK indicates experiments without chemEx3 dimer but with T-LAK cells. The data are shown as means of three experiments ± SD.

To estimate the cytotoxicity of the chemEx3 dimers against cancer cells, we applied an MTS assay in which monolayers of human bile duct carcinoma (TFK-1) cells were cultured in the presence of T-LAK cells (Figure 9). All the chemEx3 dimers showed cytotoxicity, but the half-maximal inhibitory concentrations varied. The side–tail form with a C1 linker which showed the weakest EGFR-affinity had the lowest cytotoxicity; whereas, the tail–tail form had 10 times the cytotoxicity of the side–tail form, and longer linker length promoted the cytotoxicity in each dimeric format. Consequently, the tail–tail form with a C6 linker showed a cytotoxicity 50 to 100 times that of the side–tail form with a C1 linker. This demonstrated the significance of the target affinity, direction of crosslinking, and the lengths of the intervals between scFvs in a dimer.

## 3. Discussion

In this study, we showed the potential of using lysine-free block modules for homogeneously assembling different modules to generate chimeric proteins that cannot be formed by means of genetic fusion approaches. Genetically fused chimeric proteins are directly expressed in host cells; however, the fusion sites are restricted to the N- or C-terminus of each protein, and increasing the number of fused modules increases the difficulty of expressing the chimeric proteins in host cells [35]. With chemical conjugation, coupling reagents are used to form various linkages between specific side chains of amino acids in the block modules [22], but the location and number of the amino acids used for conjugation needs to be carefully designed for homogeneous arrangement of modules. Cysteine has generally been applied for site-specific conjugation [23,24], but cysteine can induce the deactivation of the proteins, especially antibody fragments. Recently, non-natural amino acids have been utilized for site-specific conjugation [36]: proteins with non-natural acids are prepared by cell-free expression or host cells with an artificial tRNA for the amber codon, and the side chains on the non-natural amino acids are utilized for chemical conjugation. However, the position where the non-natural amino acid is induced might be restricted because it is possible that the non-natural amino acid will have a critical effect on the folding of the protein. In contrast, lysine is hydrophilic, is abundant on the surface of proteins, and this amino group has been used as a chemical conjugation site. The mutation methodology whereby lysine-free block modules are created without the deactivation of the protein enables the arrangement of the block modules in various directions with the site where the block module is to be conjugated returned to lysine again. Constructing a lysine-free module might be drawback. Actually, the case of hOTK3 scFv where the lysine-free module was created without the use of molecular evolution showed that only the bioinformatics approach was not sufficient; whereas, the results of h528 scFv showed the possibility of utilizing molecular evolution for mutation design in CDR sequence. The combination of molecular evolution and bioinformatics has the potential for reliable design of mutations for lysine-free variants. 

In the present study, the western blot experiments utilizing c-myc’ and HA-tags were applied for confirming the formation of scFv dimers with different crosslinking structures; however, the detailed characterization and homogeneity of the formed products were not sufficiently analyzed. Mass spectrometry would be useful, especially, to identify or rule out cross-reactivity of the initiator reaction to sites other than the targeted lysine side chain. Size exclusion chromatography with soluble EGFR might also supply the ratio of bivalently active forms in the formed products. In the chemical conjugation with the g-amino group in the side chain of lysine, the α-amino group at the N-terminus is possibly reacted although the a-amino group is more acidic than the γ-amino group [37]. In the present study, the change of a pair of lysine-mutated scFvs in scFv dimer showed different affinity and cytotoxicity, suggesting that the side chain of lysine residue was dominantly reacted; however, there is no critical evidence that only the γ-amino group was reacted. Recently, several specific reactions for N-terminus have been reported [38,39]. Utilization of the reaction for the capping of N-terminus would ensure the homogeneous formation of scF dimers. 

Several small T-cell-activating antibodies composed of Fvs have been reported (including diabody, single-chain diabody, and tandem single-chain formats), and it is reported that changing the structural formats results in critical differences in cytotoxicity [19,20]. For instance, in the case of diabodies, which have two scFvs with swapped heavy-chain variable (VH) and light-chain variable (VL) domains dimerized to form bispecific antibodies, changing the order of VH and VL results in a greater than 1000-fold difference in cytotoxicity [21,40,41]. The difference in domain order (HL versus LH) may result in differences in the crosslinked structure formed between the two target molecules [42,43]. The site-specific conjugation in the present study can strictly arrange two Fvs in a bivalent and bispecific antibody with an arrangement of the Fvs that cannot be formed by means of genetic fusion approaches; the tail–tail form, where the two Fvs face in opposite directions, showed higher binding affinity and cytotoxicity than did the side–tail form, where the two Fvs face in orthogonal directions. These results support the suggestion that the arrangement of the Fvs affects the binding affinity and cytotoxicity, and they show the usefulness of generating highly cytotoxic bispecific antibodies by designing the arrangement of Fvs.

In conclusion, utilization of molecular evolution and bioinformatics assisted in generating lysine-free scFvs without deactivation, and the re-mutation to lysine at one position enabled control of the arrangement of the scFvs in the chemically crosslinked bivalent and bispecific antibodies. This methodology based on the design of lysine-free block modules has the potential for the assembling functional block modules with a finely tuned structure that cannot be formed by genetic fusion approaches.

## 4. Materials and Methods

### 4.1. Expression and Purification of Recombinant Antibodies

All the gene fragments of the mutated h523 scFvs with the peptide sequence of c-myc’-tag or HA-tag, where VH domain was fused at the N-terminus of VL domain via a peptide sequence of (GGGGS)_3_, were organically synthesized, and they were ligated into the linearized pRA vectors by the digestion with *Nco*I and *Spe*I [44]; so that, the gene fragment was inserted at the 3’-side of the gene encoding the pelB signal peptide. For the hOKT3 scFv, the order of VH and VL was reversed, and the pRA vectors with the gene fragment of the mutated scFvs was prepared in the same manner as h528 scFv.

Transformed *E. coli* BL21 (DE3) were transformed with the prepared pRA encoding the scFv, and they were incubated in 2× yeast extract–tryptone medium containing 100 g/mL ampicillin at 28 °C. Expression of recombinant antibodies under the control of the T7 promoter was induced by adding 1 mM isopropyl β-d-thiogalactopyranoside at the absorbance of 0.8 at 600 nm. After additional incubation at the same condition, the harvested cells were centrifuged to separate the supernatant and pellet. From the supernatant, the wild-type scFvs were purified by means of immobilized metal-ion affinity chromatography (Ni Sepharose 6 Fast Flow; GE Healthcare Bio-Sciences AB, Uppsala, Sweden), size-exclusion chromatography (HiLoad 26/60 Superdex 200 prep grade; GE Healthcare Bio-Sciences AB), and then cation-exchange chromatography (Mono S 5/50 GL; GE Healthcare Bio-Sciences AB).

From the pellets, the lysine-mutated scFvs were refolded according to previously reported method [45]. Basically, the pellets were washed with a buffer containing Triton X-100 and they were solubilized in a buffer with 6 M GdnHCl. The solubilized scFvs were purified by means of immobilized metal-ion affinity chromatography, and the purified scFvs were refolded by gradual removal of GdnHCl by means of stepwise dialysis. After dialysis, the refolded scFvs were fractionated by means of size-exclusion chromatography.

### 4.2. Chemical Conjugation for Constructing scFv Dimers

SANH or SFB dissolved in DMSO was added into a boracic buffer (pH 8.5) containing the scFv variants and reacted for 2 h (scFv:SANH or SFB = 1:100). After removing unreacted chemical reagents by means of immobilized metal-ion affinity chromatography, the solutions were dialyzed to a phosphate buffer (pH 6.0). The solutions containing the scFv variants conjugated with SANH and the ones with SFB were equivalently mixed for 2 h, and the reacted solutions were fractionated by means of size-exclusion chromatography.

### 4.3. SPR Analysis

The interaction between soluble EGFR (sEGFR) and recombinant antibodies was examined by means of SPR spectroscopy (Biacore 2000; GE Healthcare, Chicago, IL, USA). sEGFR, which was prepared as described previously [28], was immobilized on a CM5 sensor chip in various amounts by using 50 mM sodium acetate buffer (pH 4.5) containing 20 μg/mL sEGFR. The running buffer for the experiments was PBS with 0.005% Tween20, and scFv monomer or dimer were injected for 120 s at concentrations from 10 to 250 nM. Kinetic parameters were determined by a global fitting analysis with the assumption of a 1:1 Langmuir binding model.

### 4.4. Preparation of T-LAK and Cancer Cells

CD3-expressing T-LAK cells and two cancer cells (A431: a human epidermoid carcinoma line, TFK-1: a human extrahepatic bile duct carcinoma line) cells were cultured as previously described [28]. 

### 4.5. Flow Cytometric Analysis

The specific binding of recombinant antibodies to EGFR and CD3 was assessed by flow cytometry using A431 (EGFR-positive tumor cell line) and CD3-expressing T-LAK cells. Cells (1×10^6^ per sample) were incubated on ice with scFv variants at the amounts from 0 to 2 pmol, washed with PBS, stained with wild-type scFv labeled by means of a fluorescein labeling kit (Kit-HN_2_; Dojindo Molecular Technologies, Inc., Kamimashiki, Japan), and washed again. The fluorescently labeled cells were analyzed by flow cytometry (FACSCalibur HG; BD Biosciences, Franklin Lakes, NJ, USA). To confirm ligand expression, A431 cells were incubated with the anti-mouse EGFR antibody 528, and T-LAK cells were incubated with murine anti-CD3 OTK3 IgG antibodies. Antibodies were detected by using FITC-labeled anti-mouse Fc antibodies (Thermo Fisher Scientific, Waltham, MA, USA).

### 4.6. In Vitro Cytotoxicity Assay

In vitro cytotoxicity of the recombinant antibodies was analyzed by means of an MTS assay kit (CellTiter 96 Aqueous Nonradioactive Cell Proliferation Assay; Promega, Fitchburg, WI, USA), as previously described [21]. 

The target TFK-1 cells (10,000 cells in 100 µL of culture medium) were plated in 96-well, half-area (A/2), flat-bottomed plates, and the culture medium was removed after the cells were incubated overnight. 100 µL of T-LAK effector cells and recombinant antibodies were added to each well, giving a final effector-to-target cell ratio of 2:1., and the cells were incubated for 24 h at 37 °C. The cells were washed with PBS to remove effector cells and dead target cells, and 9 µL of MTS and 0.5 µL of phenazine methosulfate solution (Promega) were added with 90.5 µL of culture medium to each well. After the incubation for 1 h at 37 °C, and the absorbance at 490 nm were measured on a microplate reader. Growth inhibition of the target TFK-1 cells was calculated as described previously [21]: percentage growth inhibition of target cells = [1 − (A_490_ of experimental sample − A_490_ of background)/(A_490_ of control sample − A_490_ of background)] × 100, where A_490_ is absorbance at 490 nm.

## Figures and Tables

**Table 1:** Mutations of lysine residues in the h528 scFv.

scFv	VH						VL				
	K position						K position				
	H12	H13	H19	H23	H62 ^a)^	H64 ^a)^	L39	L50^a)^	L74	L103	L107
h528 scFv	K	K	K	K	K	K	K	K	K	K	K
0K_CDR_	K	K	K	K	E	Q	K	Q	K	K	K
0K	A	E	Q	A	E	Q	R	Q	T	E	E

a) H62, H64, and L50 residues are located in the CDR.

**Table 2:** Surface plasmon resonance measurements for the binding affinity of h528 scFv variants with respect to EGFR.

scFv	*k*_on_ (10^4^ (Ms)^−1^)	*k*_off_ (10^−3^s^−1^)	*K*_D_ (nM)
Wild-type	19.6	5.2	26
0K_CDR_	14.1	5.4	38
0K	6.0	8.2	137
HK19-0K	6.0	7.9	135
LK103-0K	4.2	8.6	205

**Table 3:** Mutations of lysine residues in the hOKT3 scFv

scFv	VH					VL			
	K position					K position			
	H23	H43	H63 ^a)^	H65 ^a)^	H76	L41	L44	L52 ^a)^	L102
hOKT3 scFv	K	K	K	K	K	K	K	K	K
0K	A	Q	S	Q	S	Q	Q	N	E
LK52-0K	A	Q	S	Q	S	Q	Q	K	E
0K*_LK52R_*	A	Q	S	Q	S	Q	Q	R	E
LK102-0K*_LK52R_*	A	Q	S	Q	S	Q	Q	R	K

a) H63, H65, and L52 residues are located in the CDR.

**Table 4:** Surface plasmon resonance measurements for the binding affinity of scFv dimers with respect to EGFR

scFv	Immobilized EGFR (RU)	*k*_on_ (10^4^(Ms) ^−1^)	*k*_off_ (10^−3^s^−1^)	*K*_D_ (nM)
Monomer				
HK19-0K	1089	5.2	7.5	144
	2789	6.9	7.5	108
LK103-0K	1073	3.9	8.6	221
	2789	4.3	8.9	207
Dimer				
Side–Side C1	1089	4.6	3.6	78
	2833	6.4	1.9	31
Side–Side C6	1089	3.8	3.3	89
	2833	5.5	1.9	34
Side–Tail C1	1119	3.4	5.0	149
	2789	4.3	4.7	109
Side–Tail C6	1100	3.2	5.1	159
	2747	7.2	4.3	56
Tail–Tail C1	1122	2.8	2.5	88
	2605	3.1	2.0	64
Tail–Tail C6	1073	6.2	1.2	18
	2605	6.3	1.5	23

**Table 5:** Surface plasmon resonance measurement for the binding affinity of chemEx3 dimers with respect to EGFR.

scFv dimer	*k*_on_ (10^4^(Ms)^−1^)	*k*_off_ (10^−3^s^−1^)	*K*_D_ (nM)
Side–tail form			
C1	1.9	5.8	307
C6	3.8	5.8	160
Tail–tail form			
C1	1.5	5.7	380
C6	3.6	5.7	160

## Supplementary Materials

Supplementary materials can be found at https://www.mdpi.com/1422-0067/21/3/711/s1.
