# Supplementary material for: Chemically Crosslinked Bispecific Antibodies for Cancer Therapy: Breaking from the Structural Restrictions of the Genetic Fusion Approach
Source: Int J Mol Sci. 2020 Jan 21;21(3):711. doi: 10.3390/ijms21030711 (PMC7037651; doi:10.3390/ijms21030711)
Supplement: Supplementary file 1 [file ijms-21-00711-s001.pdf]

Supplementary Materials

Supplementary Table S1. Sequences of Fvs aligned with frequency. The red-colored amino acid residues are applied for 0k scFv.

(a) h528 Fv

	VH						VL				
	H12	H13	H19	H23	H62[a)]	H64[a)]	L39	L50[a)]	L74	L103	L107
Onda et al.	**A**	K,**E**	K,**Q**	K,**A**	K	K	K,**R**	T	**T**	K,**E**	K,**E**
50 - 100	V	K	K	K	S	K	K		**T**	K	K
30 - 50											
20 - 30		Q		**A**		**Q**		D	K		G
10 - 20		R	S	T	K			A<br>E<br>G			
5 - 10			R	S		R	**R**	K	N		
3 - 5		A						L<br>**Q**<br>R<br>S<br>W	S		
2 - 3	**A**<br>K<br>M			V	P	E		Y	R		
1 - 2	L			E	N<br>R<br>D	T	E	N<br>T<br>V			

(b) hOKT3 Fv

	VH					VL			
	H23	H43	H63[a)]	H65[a)]	H76	L41	L44	L52[a)]	L102
Onda et al.	K,**A**	**Q**	F	D	**S**	T	K,**Q**	**N**	K,**E**
50 - 100	K		**S**	K	**S**		K		K
30 - 50		K	K			**Q**		**N**	
20 - 30	**A**								
10 - 20	T	**Q**		**Q**	K	T<br>K	**Q**	T<br>S<br>K	
5 - 10	S	R	A<br>W		Q	G<br>S	R		
3 - 5		N<br>H			A<br>R			R	
2 - 3	V		T	R		E		Y	
1 - 2	E		N<br>P<br>D<br>R	E<br>N<br>M		A<br>F<br>N		D<br>Q<br>I	

Supplementary Table S2. Surface plasmon resonance measurement for the binding affinity of h528 scFv variants with respect to EGFR.

	k_{on} [10^4(Ms)$^{-1}$]	k_{off} [10^{-3}s^{-1}]	K_D [nM]
scFv dimer (C1)			
Side–Side	6.2	2.3	37
Side–Tail	1.4	4.0	295
Tail–Tail	3.7	2.0	63
scFv dimer (C6)			
Side–Side	6.0	1.8	30
Side–Tail	2.1	2.8	128
Tail–Tail	6.6	1.6	14

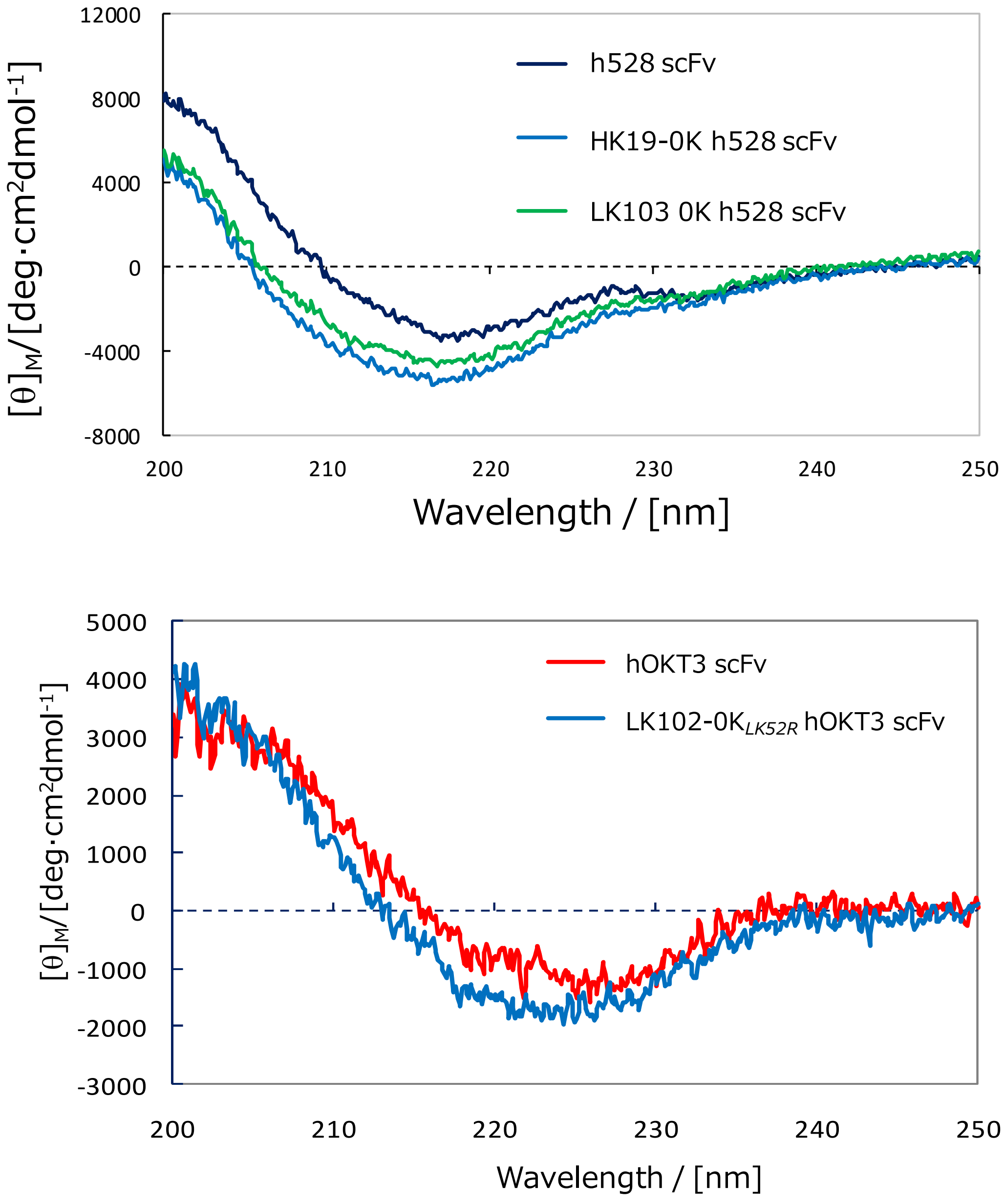

Supplementary Figure S1. CD spectra of h528 scFv, HK19-0K h528 scFv, LK103-0K h528 scFv, hOKT3 scFv, and LK102-0K$_{LK52R}$ hOKT3 scFv.

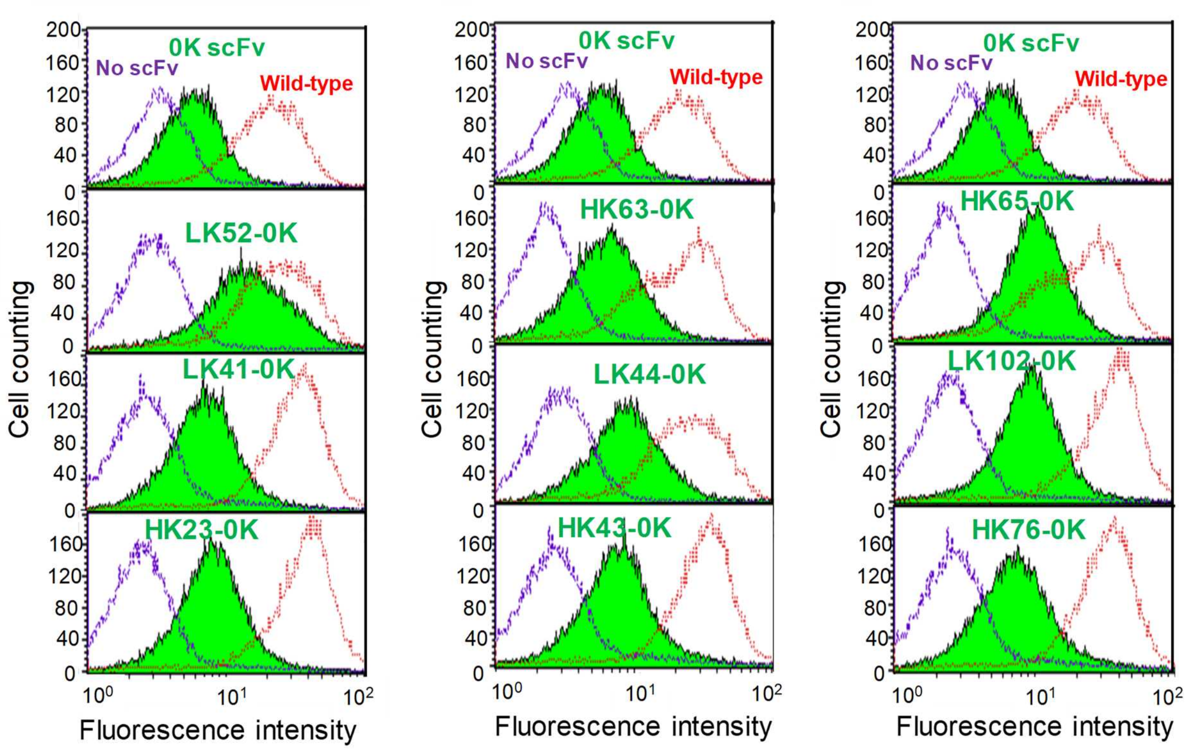

Supplementary Figure S2. Flow cytometry histograms of T-LAK cells measured with flow cytometry. 10^6 CD3-expressing T-LAK cells were incubated with each hOKT3 scFv variant (3 nmol), washed with PBS, and stained with FITC-labeled anti-c-myc' IgG antibody. The fluorescently labeled cells were then analyzed by means of flow cytometry (filled green). Cells incubated with hOKT3 wild-type scFv were also analyzed by FITC-labeled anti-c-myc' IgG antibody (red). The results for cells incubated without scFv are navy blue.

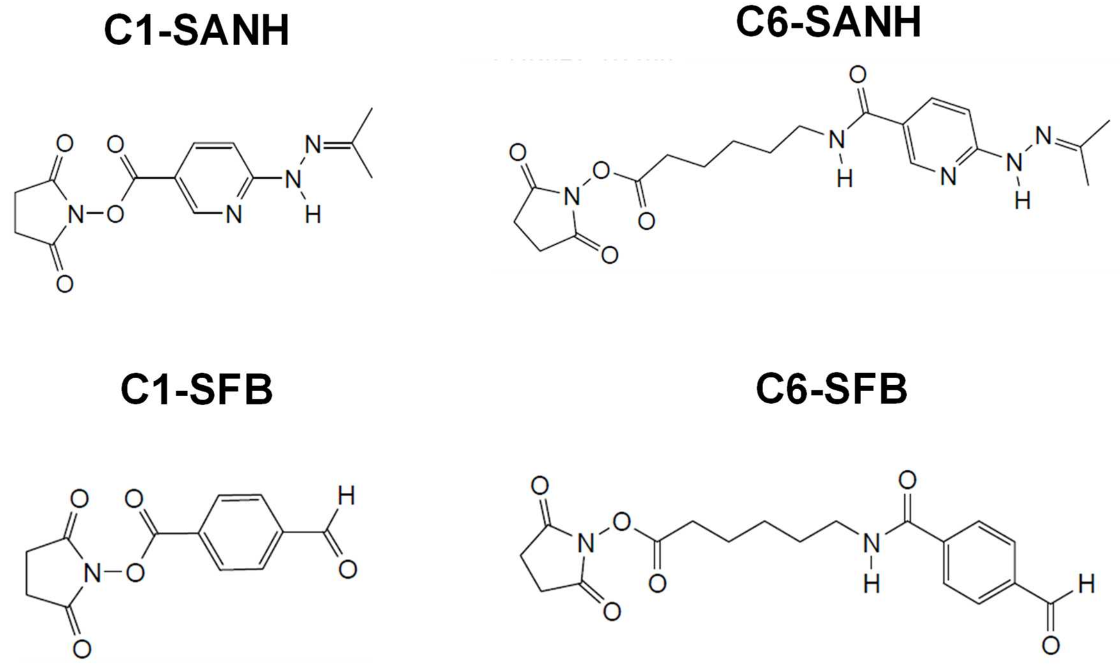

Supplementary Figure S3. Chemical structure of initiator molecules

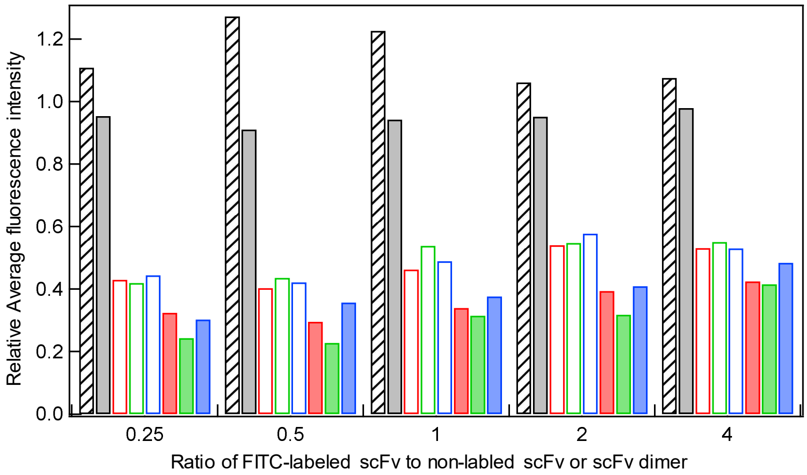

Supplementary Figure S4. Competitive binding assay of non-labeled HK19-0K scFv (striped), HK-19-0K or LK103-0K scFv (gray), side–side dimers with C1 and C6 linkers (open and closed red), side–tail dimers with C1 and C6 linkers (open and closed green), and tail–tail dimers with C1 and C6 linkers (open and closed blue), with FITC-labeled wild-type scFv. Average fluorescence intensity was normalized by the competitive binding assay of non-labeled wild-type scFv with FITC-labeled wild-type scFv. A431 cells were used.

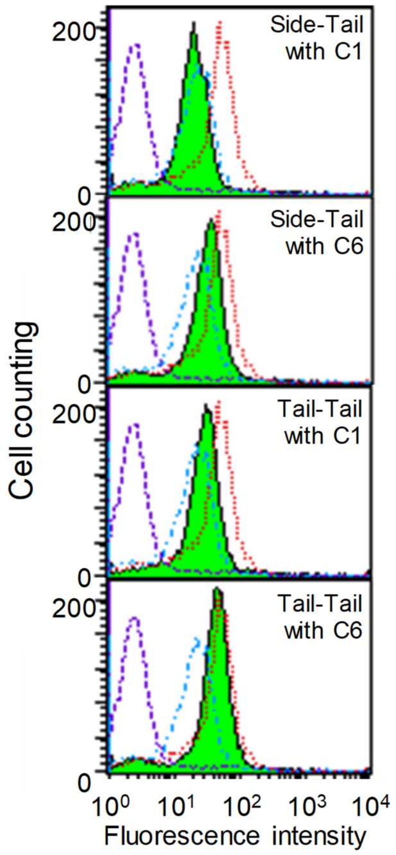

Supplementary Figure S5. Flow cytometry histogram of T-LAK cells measured with flow cytometry. 10^6 cells were incubated with chemEx3 dimer labeled with FITC-labeled anti-c-myc′ IgG antibody and then analyzed by means of flow cytometry (filled green). Cells incubated with hOKT3 wild-type and LK102-0K scFv were also analyzed by FITC-labeled anti-c-myc′ IgG antibody (red and light blue, respectively). The results of the cells without scFv or chemEx3 dimer are navy blue.
